# Supplementary figures and images for: Circadian programming of the ellipsoid body sleep homeostat in Drosophila
Source: eLife. 2022 Jun 23;11:e74327. doi: 10.7554/eLife.74327 (PMC9270026; doi:10.7554/eLife.74327)

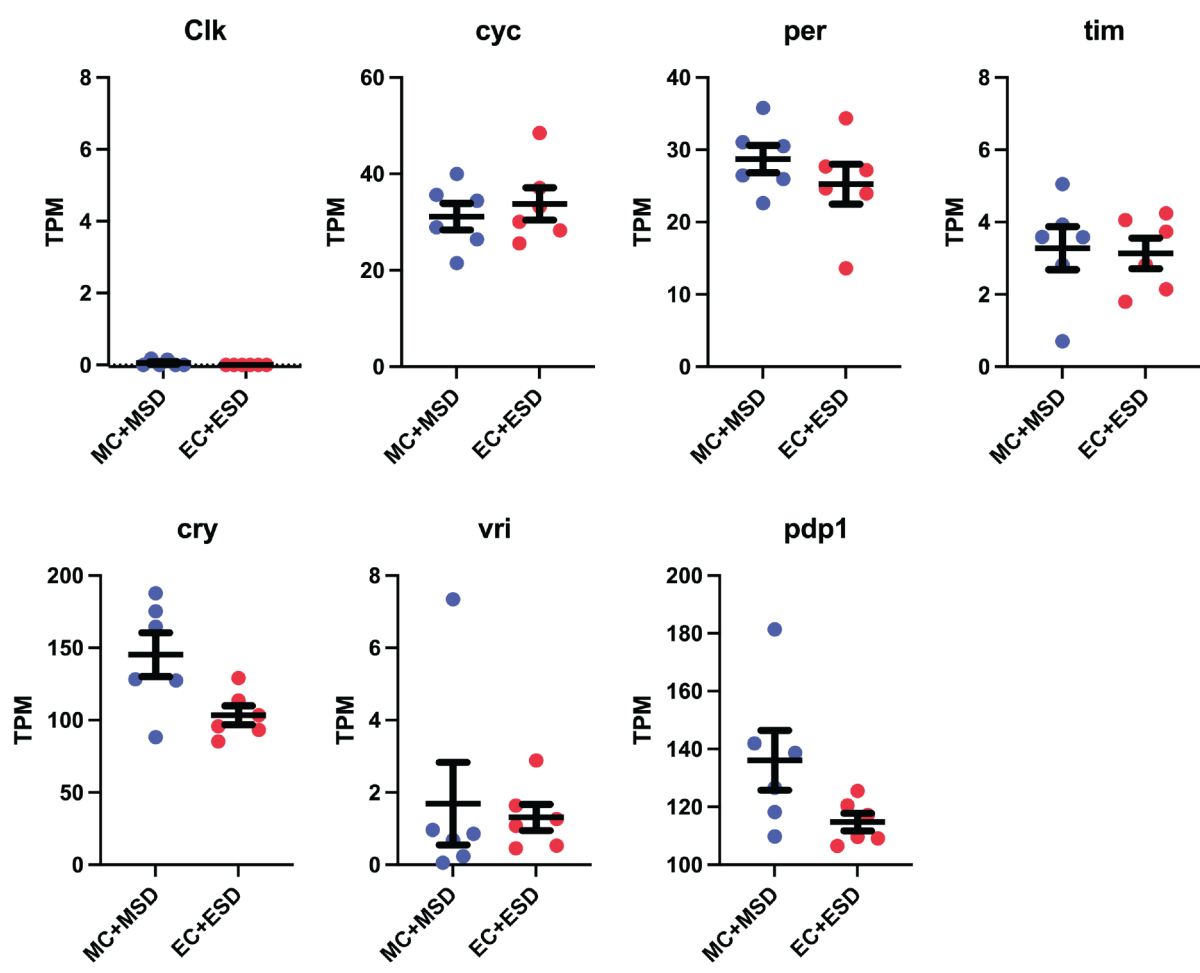

Clk

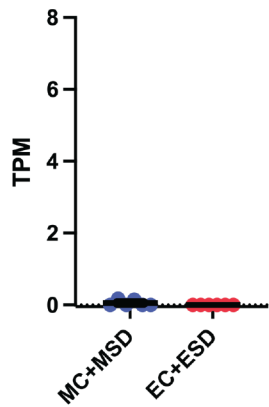

cyc

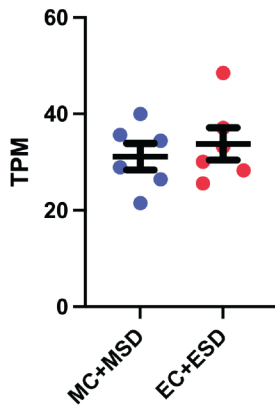

per

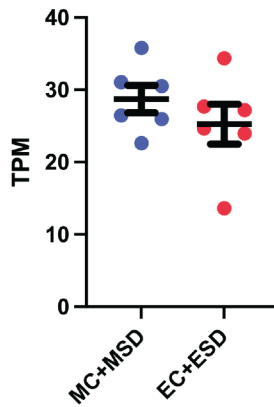

tim

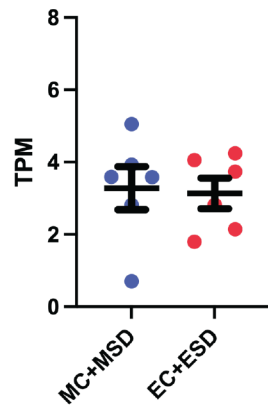

cry

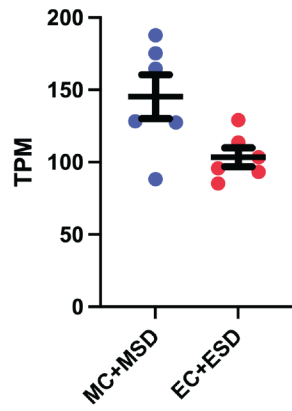

vri

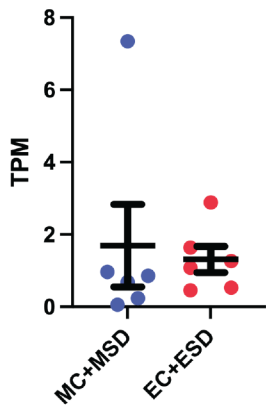

pdp1

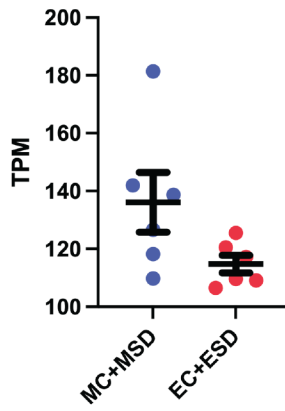

Supplement: Figure 8—figure supplement 1—source data 1. [file elife-74327-fig8-figsupp1-data1.pdf]
